# Integrated transcriptomic and metabolomic analysis reveals key regulatory genes and pathways associated with feed conversion efficiency in Tianchang Sanhuang chicken

**DOI:** 10.1016/j.psj.2025.105912

**Published:** 2025-09-27

**Authors:** Jiale Li, Shenghe Li, Mengmeng Zhuansun, Xinyu Liu, Tao Jin, Kefeng Yang, Man Ren, Erhui Jin, Xiaojin Li, Mengmeng Jin, Chunfang Zhao

**Affiliations:** a College of Animal Science, Anhui Science and Technology University, Chuzhou 233100, PR China; b Anhui Province Key Laboratory of Animal Nutritional Regulation and Health, Chuzhou 233100, PR China; c Anhui Engineering Technology Research Center of Pork Quality Control and Enhance, Hefei 236065, PR China

**Keywords:** Residual feed intake, Tianchang Sanhuang chicken, Transcriptome, Metabolome, Key signaling pathway

## Abstract

Improving feed efficiency in Tianchang Sanhuang chickens is essential for reducing production costs and environmental burden. The objective of this study was to integrate transcriptomic and metabolomic analyses to identify key regulatory genes, metabolites, and pathways associated with residual feed intake (RFI) and feed efficiency. In this study, 650 Tianchang Sanhuang laying hens with similar body weights at 36 weeks of age were evaluated for daily feed intake (DFI), RFI, and feed conversion ratio (FCR). The chickens were classified by RFI (mean ± 0.5 SD) into high-RFI (HRFI, *n* = 165) and low-RFI (LRFI, *n* = 158) groups. Phenotypes, serum biochemistry, antioxidant indices, and intestinal traits were compared in subsets (*n* = 8 per group). Duodenal transcriptomes (RNA-seq) and serum metabolomes (LC-MS/MS) were profiled in independent subsets (*n* = 4 per group). Compared with HRFI, LRFI hens showed lower RFI, FCR, and DFI (*P* < 0.01), with no differences in expected feed intake (EFI), metabolic body weight (MBW), daily egg mass (DEM), or average daily gain (ADG) (*P* > 0.05). The LRFI group showed increased breast muscle redness (a)* (*P* < 0.05), higher leg muscle drip loss (*P* < 0.01), and significantly lower levels of triglycerides (TG), cholesterol (CHO), low-density lipoprotein cholesterol (LDL-C), and malondialdehyde (MDA) (*P* < 0.05). The intestinal morphology and molecular analyses revealed enhanced nutrient absorption and intestinal barrier function in the LRFI group. Transcriptomic analysis identified 237 differentially expressed genes (*P* < 0.05, |log2FC| ≥ 1) enriched in pathways related to digestion, energy metabolism, and appetite regulation. Metabolomic analysis detected 101 differentially expressed metabolites (VIP ≥ 1, |log2FC| ≥ 1), indicating that RFI is closely associated with protein and lipid metabolism. Integrated analysis identified candidate biomarkers for low RFI individuals selection, including genes such as *ACSM5, AHSG, CTRB1, PLA2G1B, AMY2A, CPA1, CCKAR* and metabolites including taurine, uridine, L-phenylalanine, D-glucose 6-phosphate and 5‑hydroxy-L-tryptophan. Overall, LRFI hens maintain production while achieving lower intake, potentially via reduced inflammation/oxidative stress and enhanced digestion, barrier integrity, appetite, and energy metabolism, offering targets for marker-assisted improvement of feed efficiency in local breeds.

## Introduction

Feed costs account for 65–70% of the total production expenses in poultry farming, and this proportion continues to rise, especially under the current trend of continuously increasing prices of feed ingredients (Mottet and Tempio, 2017). Improving feed efficiency is one of the key objectives in current poultry breeding, as it helps reduce feed costs. Currently, the indicators used to evaluate feed efficiency include FCR and RFI. FCR is defined as the ratio of feed consumption to output over a given period of time (Hess et al., 1941). In laying hens, it is expressed as the feed-to-egg ratio. Its main advantage lies in its simplicity and ease of calculation. However, studies have shown that FCR, as a ratio trait, exhibits a non-normal distribution within populations and has a low genetic correlation with DFI (Aggrey et al., 2010). Therefore, FCR is not considered an ideal indicator for selecting traits associated with high feed efficiency. The concept of RFI was first introduced by Koch et al. (1963) in beef cattle production, and it is defined as the difference between an animal’s actual feed intake and the expected feed intake required for maintenance and production. A lower RFI value (RFI < 0) indicates higher feed efficiency, whereas a higher RFI value (RFI > 0) suggests that the animal’s feed intake exceeds the expected intake required for maintenance and production. Studies have shown that RFI in laying hens has moderate heritability (*h²* = 0.52) and exhibits low correlations with other economic traits (Zhou et al., 2022). Therefore, selection based on RFI can effectively improve feed efficiency in poultry without negatively affecting production performance.

Currently, the researches on RFI primarily focus on elucidating its molecular regulatory mechanisms and identifying markers for molecular-assisted selection. Transcriptome sequencing of chickens with divergent RFI values revealed that LRFI birds may enhance feed efficiency by reducing energy expenditure associated with cellular activity, immune responses, and physical activity (Xu et al., 2016). Based on transcriptomic data, Xiao et al. (2021) identified that gene expression in male LRFI chickens is associated with the structure and function of intestinal microvilli, as well as lipid metabolic processes. In contrast, gene expression in female LRFI chickens is primarily related to the nervous system and cellular development. In addition, metabolomic analysis of the rumen in beef cattle has revealed significant associations between RFI and pathways related to protein digestion and absorption, linoleic acid metabolism, lysine degradation, and fatty acid degradation (Liu et al., 2022). Transcriptomics and metabolomics are powerful tools for characterizing changes in mRNA and metabolites under different RFI levels. The integration of transcriptomic and metabolomic analyses facilitates a deeper understanding of the molecular mechanisms underlying variations in RFI, thereby providing more precise and effective guidance for breeding programs.

Tianchang Sanhuang chicken is an important dual-purpose indigenous breed from Anhui Province, China, valued for both egg and meat production. It is well known for its tender meat, distinctive flavor, and adaptability to local farming conditions. However, compared with commercial breeds, Sanhuang chickens exhibit lower feed conversion ratio, requiring more feed to achieve comparable egg production or body weight gain (Lv et al., 2023). Although previous studies have explored feed efficiency in other Chinese indigenous chicken breeds, the Tianchang Sanhuang chicken has unique genetic traits, adaptability, and environmental resilience. Its specific genetic background and physiological characteristics may lead to distinct regulatory pathways affecting feed efficiency. Understanding these mechanisms is essential for improving feed efficiency without compromising production performance, especially in small-scale and local farming systems.

In addition to production performance, feed efficiency may affect other economically important traits such as meat quality, physiological metabolism, oxidative stress, and intestinal function. Meat quality can be influenced by nutrient partitioning and energy utilization efficiency, and evaluating these traits in high and low-RFI chickens can provide insights into overall resource allocation strategies (Mir et al., 2017). Moreover, blood biochemical and antioxidant indices reflect metabolic and oxidative stress level (Yang et al., 2020b), while intestinal morphology and barrier integrity directly influence nutrient absorption and utilization (Khan and Islam, 2012). Understanding these parameters in the context of RFI variation is essential for comprehensive evaluation and potential breeding strategies. We hypothesize that Tianchang Sanhuang chickens exhibit distinct genetic and metabolic regulatory pathways for feed efficiency that may differ from those identified in other breeds. These pathways are likely to involve digestive enzymes, lipid metabolism, gut health, and oxidative stress, which are critical for improving feed efficiency in dual-purpose breeds. In this study, transcriptomic and metabolomic profiling of Tianchang Sanhuang laying hens with divergent RFI values is conducted to identify key differentially expressed genes (DEGs) and metabolites (DEMs) and assess their roles in feed efficiency. The primary goal of this study is to improve feed efficiency in laying hens, while also examining its correlation with meat quality traits to ensure the findings are relevant for enhancing overall resource utilization.

## Materials and methods

### Experimental animals and treatments

A total of 650 Tianchang Sanhuang laying hens (36 weeks of age, female) with similar body weights were randomly selected as experimental individuals. All chickens were housed individually in cages measuring 45*34*32 cm^3^ (length*width*height). Each cage was equipped with an independent feeder, and all individuals were provided with the same diet. The composition and nutritional value of the feed were shown in Supplementary Table S1. The protocol for using the chicken was reviewed and approved by the Anhui Laboratory Animal Care Committee (experimental approval number AHSTULL2023021). All animal experimental procedures strictly adhered to the Laboratory Animal Care and Use guidelines and complied with the National Guide for Laboratory Animal Healthcare and Use standards.

### Data collection and RFI calculation

Feed was weighed daily and manually added to each feed trough. The residual feed was weighed on the following day. Feed intake (FI) was calculated as the difference between the amount of feed added and the amount of feed remaining. The initial body weight (BW1) and final body weight (BW2) of each hen were recorded at the beginning and end of the experimental period, respectively. Additionally, the number of eggs laid and the corresponding egg weights were recorded for each hen throughout the experiment. Based on the above measurements, the DFI, MBW, DEM, ADG, and FCR were calculated.

After calculation, hens that did not lay any eggs were excluded from further analysis. Outliers for each parameter were identified and removed using the criterion of values falling outside the range of mean ± 2 SD.

RFI was calculated for the remaining individuals based on the method described by Luiting and Urff (1991), using the following equations:

(1) $$EFI=a+b_{1}*ADG+b_{2}*MBW+b_{3}*DEM$$

(2) RFI=DFI−EFI

The EFI values were obtained using a multiple linear regression model implemented in Microsoft Excel. In this model, a represents the intercept, and b₁, b₂, and b₃ are the partial regression coefficients corresponding to ADG, MBW, and DEM, respectively.

Finally, the chickens were classified into HRFI and LRFI groups based on the following criteria: RFI ≥ mean + 0.5 SD for the HRFI group, and RFI ≤ mean − 0.5 SD for the LRFI group.

### Analysis of meat quality traits

A total of 8 chickens per group (HRFI, *n* = 8; LRFI, *n* = 8) were selected from the extreme ends of the RFI distribution for meat quality measurement. All selected chickens were fasted for 12 h with free access to water, and then euthanized by cervical dislocation. After slaughter, the left-sided breast and leg muscles were separated. The following traits were measured: muscle pH, meat color, cooking loss, drip loss, water-holding capacity, and shear force.

Muscle pH was measured in breast and leg samples using a portable pH meter (S220, Mettler Toledo, Switzerland). Color parameters (L*, a*, and b*) were recorded using a CR-410 colorimeter (Konica Minolta, Japan). Drip loss was determined on approximately 1 cm³ muscle cubes suspended at 4°C for 24 h, and cooking loss was calculated from weight differences before and after steaming for 30 min; both losses were expressed as percentages of initial weight. Cooked-meat tenderness was assessed with an RH—N50 meat tenderness meter (RunHu Instruments, China). Water-holding capacity of fresh muscle was evaluated by pressing samples at 343 N for 5 min using an RH-1000 meat press (RunHu Instruments, China) and comparing pre- and post-press weights. All assays were performed in triplicate, and mean values were reported.

### Analysis of serum biochemical parameters

Blood samples were collected from the HRFI (*n* = 8) and LRFI (*n* = 8) groups before slaughter. A 4-5 mL blood sample of each chicken was collected from a wing venipuncture and centrifuged at 3,000 rpm for 15 min at 4°C to obtain serum. The concentrations of alanine aminotransferase (ALT), alkaline phosphatase (ALP), total protein (TP), albumin (ALB), globulin (GLB), triglycerides (TG), cholesterol (CHO), high-density lipoprotein cholesterol (HDL-C), and low-density lipoprotein cholesterol (LDL-C) were measured using an automated biochemistry analyzer (ZUOYUE450, Shanghai Kehua Bio-Engineering, China).

### Analysis of antioxidant parameters

Serum samples (HRFI, *n* = 8; LRFI, *n* = 8) were centrifuged at 3,500 rpm for 10 min at 4°C to obtain the supernatant for antioxidant parameter analysis. The following parameters were measured using kits from Nanjing Jiancheng Bioengineering Institute for total antioxidant capacity (T-AOC), catalase (CAT), glutathione peroxidase (GSH-PX), total superoxide dismutase (T-SOD), and malondialdehyde (MDA).

### Histological analysis of duodenal morphology

Duodenal tissues were fixed in 4% paraformaldehyde for 24 h and then trimmed into 2 × 3*0.5 mm³ blocks. The tissue blocks were rinsed in running water for 12 h to remove residual fixative. The blocks were then subjected to a graded dehydration process: overnight treatment with 70% ethanol, followed by 3 h in 80% ethanol, 2 h in 90% ethanol, 2 h in 95% ethanol I, 1 h in 95% ethanol II, 1 h in 100% ethanol I, and 1 h in 100% ethanol II. After dehydration, the samples were cleared in benzene for 20 min, then treated with xylene I for 10 min and xylene II for 10-20 min. The samples were infiltrated with paraffin I and paraffin II at 60°C for 1 h each before embedding in paraffin. The tissue sections were cut to a thickness of 5 µm. The sections were stained with hematoxylin-eosin (H&E) and mounted with neutral resin. Finally, histological observations were performed using a Motic B3 microscope (Motic Instruments Inc., USA).

### Quantitative real-time PCR (qRT-PCR) analysis for mRNA expression levels

Gene expression was quantified by qRT-PCR on an ABI Prism 7500 HT using SYBR Premix Ex Taq II (TaKaRa, Dalian, CHINA) with cycling at 95°C for 15 min, then 40 cycles (95°C for 10 s, 60°C for 32 s). Primers designed in Primer Premier 5.0 from NCBI chicken cDNA (Supplementary Table S2) were used with *GAPDH* as the internal control. Relative expression (mean ± SE) was calculated by the 2^−ΔΔCt^ method.

### Total RNA extraction and RNA-Seq analysis

Duodenal samples were collected within 20 min post-slaughter (HRFI, *n* = 4; LRFI, *n* = 4), with all samples taken from the same anatomical location at the anterior end of the duodenum. The collected tissues were immediately frozen in liquid nitrogen and stored at −80 °C for subsequent analysis.

Total RNA was isolated with TRIzol (Invitrogen) and quality‐controlled by agarose gel electrophoresis, Qubit fluorometry, NanoPhotometer (OD260/280, OD260/230) and Agilent 2100 Bioanalyzer analysis. Poly(A) mRNA was captured with oligo(dT) beads, fragmented, and reverse-transcribed into first-strand cDNA using random hexamers and M-MuLV reverse transcriptase. After RNase H treatment and second‐strand synthesis with DNA polymerase I, the double-stranded cDNA was end-repaired, A-tailed and adapter-ligated. Fragments of approximately 200 bp were size-selected with AMPure XP beads, PCR-amplified, purified again, and the final libraries were sequenced on an Illumina platform (Genedenovo, Guangzhou, China).

### Data quality control and alignment analysis

To ensure data quality, raw reads were processed using fastp for quality control. Reads containing adapters, reads with more than 10% undefined bases (N), reads consisting entirely of adenine (A), and low-quality reads (those in which bases with a quality score ≤ 20 accounted for more than 50% of the read) were removed to obtain high-quality clean reads. After filtering, the base composition and quality distribution of the reads were analyzed. Clean reads were first aligned to the chicken ribosomal RNA database using Bowtie2, and reads that aligned to rRNA were removed without allowing mismatches. The remaining unmapped reads were retained for downstream transcriptomic analysis. Clean reads were then aligned to the chicken reference genome GRCg6a (version: GCA_000002315.5) using HISAT2.

### Differential gene expression analysis and functional annotation

Gene expression was quantified as FPKM, and DESeq2 was used to identify DEGs (*P* < 0.05, |log_2_FC| ≥ 1). Enrichment analysis was performed with GOATOOLS for Gene Ontology terms and via the KEGG database for pathway annotation.

### Construction of DEGs interaction network and identification of hub genes

A protein-protein interaction (PPI) network of DEGs was built in STRING v11.5 (confidence ≥ 0.4, experimental and text-mined interactions) and imported into Cytoscape 3.8.0. Using the CytoHubba plugin, hub genes were identified by MNC, MCC, Degree, and EPC analyses; the top 10 from each metric were combined to form the final hub gene set.

### Untargeted metabolomics analysis

Eight serum samples (HRFI, *n* = 4; LRFI, *n* = 4) were thawed at 4°C, and 100 µL was precipitated with 400 µL cold methanol/acetonitrile (1:1 v/v). After vortexing and −20°C incubation for 30 min, samples were centrifuged (14,000×*g*, 20 min, 4°C), and supernatants were vacuum-dried. Dried extracts were reconstituted in 100 µL acetonitrile/water (1:1 v/v), vortexed, centrifuged (14,000×*g*, 15 min, 4°C), and the final supernatants were subjected to LC-MS.

Samples were separated on an Agilent 1290 Infinity UHPLC with a HILIC column (25°C, 0.5 mL/min, 2 µL injection). Mobile phase A was water with 25 mM ammonium acetate/ammonia; B was acetonitrile. The gradient ran: 0-0.5 min at 95% B; 0.5-7 min down to 65% B; 7-8 min down to 40% B; 8-9 min hold at 40% B; 9-9.1 min up to 95% B; 9.1-12 min hold at 95% B. Samples were kept at 4°C in the autosampler, analyzed in randomized order, and interspersed with QC injections to ensure system stability.

Raw data were converted to mzML using ProteoWizard (v3.0.6428) and processed in XCMS (online v3.7.1) using centWave peak picking (10 ppm, peakwidth = 10-60, prefilter = 10,100) and grouping (bw = 5, mzwid = 0.025, minfrac = 0.5). After feature extraction, ions with > 50% missing values were removed, remaining gaps imputed by KNN, and features with RSD > 50% excluded. The cleaned dataset was then used for metabolite identification and further statistical analyses. Metabolic differences between HRFI and LRFI groups were assessed by PLS-DA and OPLS-DA; metabolites with VIP > 1 and *P* < 0.05 were deemed significant and subjected to KEGG pathway enrichment (pathways with *P* < 0.05 considered enriched).

### Integrated analysis of transcriptomic and metabolomic data

Genes with SD ≤ 0.5 were removed, and a weighted co-expression network was built in WGCNA (v1.70-3) using blockwiseModules (power = 10, minModuleSize = 30, mergeCutHeight = 0.25). Modules with |GS| ≥ 0.5 and |MM| ≥ 0.7 were retained. Pearson correlations linked module eigengenes to key metabolites. Joint KEGG enrichment and DEGs-DEMs Pearson correlation analyses identified significant gene-metabolite pairs.

## Results

### Production Performance and correlation analysis

**Fig. 1:**
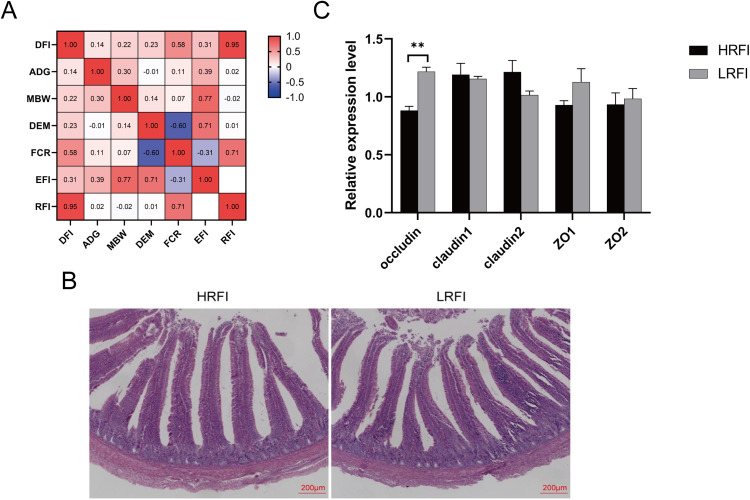
Phenotypic and intestinal differences between high and low RFI Tianchang Sanhuang chickens. A. Correlation analysis between RFI and different production traits. B. Histological sections of duodenal morphology in different RFI groups. C. Relative mRNA expression levels of duodenal tight junction proteins in different RFI groups.

Compared with the HRFI group, the LRFI chickens exhibited markedly lower RFI, FCR, and DFI (*P* < 0.01), while growth- and production-related traits such as EFI, MBW, DEM, and ADG remained unchanged (*P* > 0.05) (Table 1). Correlation analysis revealed that RFI was strongly associated with DFI (R² = 0.95) and moderately correlated with FCR (R² = 0.71), suggesting that reduced feed intake was the primary driver of improved feed efficiency in LRFI chickens (Fig. 1A). Importantly, no significant change in ADG and DEM indicates that improved efficiency was achieved without compromising growth or egg production.

**Table 1:** Production performance of Tianchang Sanhuang chickens in different RFI groups.

Items	HRFI	LRFI	*P*-value
RFI(g/d)	18.50 ± 0.55^A^	−20.10 ± 0.70^B^	< 0.01
FCR(g/g)	3.93 ± 0.07^A^	2.72 ± 0.05^B^	< 0.01
EFI(g/d)	105.73 ± 0.51	104.99 ± 0.56	0.33
DFI(g/d)	124.23 ± 0.65^A^	84.89 ± 0.88^B^	< 0.01
MBW(g)	243.81 ± 1.79	242.22 ± 1.95	0.55
DEM(g/d)	32.74 ± 0.45	32.27 ± 0.51	0.49
ADG(g/d)	2.23 ± 0.26	1.90 ± 0.30	0.40

Note: Values are presented as means ± standard error (SE).

^a–b^ Different lowercase superscript letters within the same row indicate statistically significant differences (*P* < 0.05), while ^A–B^ different uppercase letters indicate highly significant differences (*P* < 0.01). The same annotation rules apply to all tables.

### Muscle quality and meat traits

Analysis of muscle quality parameters revealed that the LRFI group exhibited a significantly higher breast muscle redness a* (*P* < 0.05) and greater drip loss in leg muscle (*P* < 0.01) compared with the HRFI group (Table 2). Although other traits such as pH, shear force, and cooking loss did not differ significantly, these subtle differences suggest that improved feed efficiency may be accompanied by altered energy allocation, influencing muscle color and water-holding capacity.

**Table 2:** Muscle quality of Tianchang Sanhuang chickens in different RFI groups.

Items		HRFI	LRFI	*P*-value
pH	Breast muscle	6.23 ± 0.09	6.40 ± 0.12	0.28
	Leg muscle	6.70 ± 0.06	6.69 ± 0.09	0.92
Lightness (L)*	Breast muscle	59.66 ± 1.46	63.02 ± 2.33	0.24
	Leg muscle	57.84 ± 2.83	55.67 ± 1.14	0.49
Redness (a)*	Breast muscle	9.51 ± 0.93ᵇ	12.79 ± 0.99ᵃ	0.03
	Leg muscle	15.80 ± 1.38	16.83 ± 1.25	0.59
Yellowness (b)*	Breast muscle	11.53 ± 0.73	12.16 ± 1.50	0.71
	Leg muscle	7.34 ± 1.00	9.25 ± 1.00	0.20
Cooking loss (%)	Breast muscle	34.36 ± 0.39	34.77 ± 1.22	0.75
	Leg muscle	42.43 ± 0.36	41.84 ± 0.82	0.52
Drip loss (%)	Breast muscle	14.78 ± 1.81	13.37 ± 2.45	0.65
	Leg muscle	15.76 ± 2.19^B^	26.81 ± 2.86^A^	< 0.01
Water-holding capacity (%)	Breast muscle	37.77 ± 0.76	38.33 ± 0.76	0.61
	Leg muscle	31.77 ± 0.99	29.27 ± 3.77	0.53
Shear force (N)	Breast muscle	15.77 ± 1.34	15.92 ± 1.73	0.95
	Leg muscle	21.43 ± 1.98	21.06 ± 1.76	0.89

### Blood biochemistry and antioxidant status

Biochemical analysis showed that LRFI chickens had significantly lower serum TG, CHO, and LDL-C levels (*P* < 0.05), indicating more efficient lipid utilization (Table 3). Consistently, oxidative stress marker MDA was also lower in the LRFI group (*P* < 0.05), while antioxidant enzyme activities (CAT, GSH-PX, and T-SOD) remained unchanged (Table 4). These findings suggest that improved feed efficiency is associated with reduced lipid accumulation and alleviated oxidative damage.

**Table 3:** Blood biochemical parameter concentrations of Tianchang Sanhuang chickens in different RFI groups.

Items	HRFI	LRFI	*P*-value
ALT (U/L)	3.88 ± 0.76	4.27 ± 0.47	0.68
ALP (U/L)	397.26 ± 59.41	497.78 ± 96.13	0.41
TP (g/L)	53.84 ± 5.21	54.66 ± 4.87	0.91
ALB (g/L)	22.45 ± 2.16	21.46 ± 1.34	0.71
GLB (g/L)	31.39 ± 3.24	33.20 ± 5.44	0.79
TG (mmol/L)	13.70 ± 0.73^a^	8.79 ± 1.20^b^	0.01
CHO (mmol/L)	3.60 ± 0.19^a^	2.62 ± 0.27^b^	0.03
HDL-C (mmol/L)	0.50 ± 0.04	0.65 ± 0.05	0.06
LDL-C (mmol/L)	1.12 ± 0.08^a^	0.79 ± 0.08^b^	0.02

**Table 4:** Serum antioxidant levels of Tianchang Sanhuang chickens in different RFI groups.

Items	HRFI	LRFI	*P*-value
T-AOC (U/mL)	0.17 ± 0.00	0.16 ± 0.01	0.62
CAT (U/mL)	25.94 ± 1.34	26.32 ± 0.92	0.82
GSH-PX (U/mL)	112.98 ± 28.17	102.26 ± 24.78	0.79
T-SOD (U/mL)	10.88 ± 0.42	10.28 ± 0.61	0.45
MDA (nmol/mL)	4.33 ± 0.45^a^	2.75 ± 0.46^b^	0.04

### Duodenal morphology and tight junction gene expression

Histological analysis revealed that LRFI chickens had shallower crypt depth (CD) and higher VH/CD (V/C) ratios (*P* < 0.05), together with higher expression of the tight junction protein *Occludin* (*P* < 0.05) (Table 5, Fig. 1B, 1C). These results indicate that LRFI chickens may possess a more efficient intestinal barrier, which likely facilitates enhanced nutrient absorption while reducing metabolic burden.

**Table 5:** Duodenal morphological parameters of Tianchang Sanhuang chickens in different RFI groups.

Items	HRFI	LRFI	*P*-value
VH(µm)	975.06 ± 51.27	979.41 ± 43.51	0.95
CD(µm)	214.02 ± 11.37^A^	166.96 ± 4.15^B^	< 0.01
V/C	4.60± 0.38^b^	5.87 ± 0.20^a^	0.03

### Correlation analysis between RFI and significantly associated phenotypic traits

The correlation analysis revealed that RFI was significantly positively correlated with TG, CHO, LDL-C, and MDA (*P* < 0.05), indicating that high-RFI chickens exhibited elevated blood lipid levels and increased oxidative stress (Table 6). In contrast, RFI was significantly negatively correlated with *Occludin* expression and V/C (*P* < 0.01) and positively correlated with CD (*P* < 0.01), suggesting that low-RFI chickens had better intestinal barrier integrity and enhanced nutrient absorptive capacity. Regarding meat quality traits, RFI was significantly negatively correlated with breast muscle redness a* and leg muscle drip loss (*P* < 0.05), indicating that low-RFI chickens exhibited redder breast muscle color but slightly poorer leg muscle water-holding capacity. Collectively, these findings demonstrate that RFI in Tianchang Sanhuang chickens is closely associated with lipid metabolism, oxidative stress, intestinal morphology, and meat quality traits.

**Table 6:** Phenotypic correlations between RFI and significantly associated traits.

Items	RFI	Redness (a)*	Drip loss	TG	CHO	LDL-C	MDA	CD	V/C	*Occludin*
RFI	1	−0.56*	−0.62**	0.72**	0.64**	0.63**	0.59*	0.81**	−0.75**	−0.78**
Redness (a)*		1	0.46	−0.41	−0.45	−0.52*	−0.63**	−0.53*	0.33	0.45
Drip loss			1	−0.48	−0.40	−0.51*	−0.50*	−0.55*	0.51*	0.65**
TG				1	0.92**	0.86**	0.43	0.71**	−0.64**	−0.62*
CHO					1	0.89**	0.42	0.65**	−0.57*	−0.67**
LDL-C						1	0.38	0.68**	−0.56*	−0.75**
MDA							1	0.55*	−0.53*	−0.62**
CD								1	−0.82**	−0.72**
V/C									1	0.67**
*Occludin*										1

Note: * indicates a statistically significant correlation (*P* < 0.05), and ** indicates a highly significant correlation (*P* < 0.01).

### Duodenal transcriptomics and differential expressed genes analysis

**Fig. 2:**
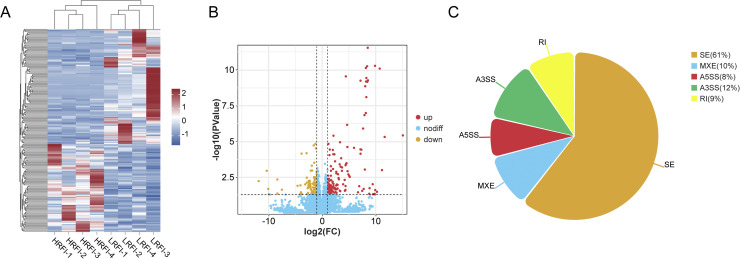
Differential analysis of transcriptomes between high and low RFI groups. A. Heatmap of differentially expressed genes. B. Volcano plot of DEGs. C. Differential alternative splicing analysis.

After quality control, the proportion of clean reads in all samples exceeded 99.41%, with Q20 and Q30 values above 97.36% and 92.93%, respectively, indicating reliable quality of the raw data (Supplementary Table S3). The GC content was stable (50.08%–52.79%), and genome alignment rates ranged from 86.61% to 92.68%, which met the requirements for subsequent bioinformatics analysis. A total of 17,868 expressed genes were detected. Differential expression analysis identified 237 DEGs between LRFI and HRFI groups (|log₂FC| ≥ 1, *P* < 0.05), including 129 upregulated and 108 downregulated in the LRFI group (Supplementary Table S4). Hierarchical clustering (Fig. 2A) and volcano plot visualization (Fig. 2B) revealed distinct separation between the two groups. The top 10 upregulated and downregulated genes are presented in Supplementary Table S5. The validation of eight randomly selected DEGs by RT-qPCR confirmed the accuracy of RNA-seq data (Fig. 4C, D). In addition, alternative splicing analysis showed notable differences between groups, with five major types of events detected (Fig. 2C). They included skipped exon (SE, 61%), mutually exclusive exon (MXE, 10%), alternative 5′ splice site (A5SS, 8%), alternative 3′ splice site (A3SS, 12%), and retained intron (RI, 9%). These results suggest that exon skipping represents the predominant regulatory mechanism of alternative splicing in divergent RFI groups.

**Fig. 3:**
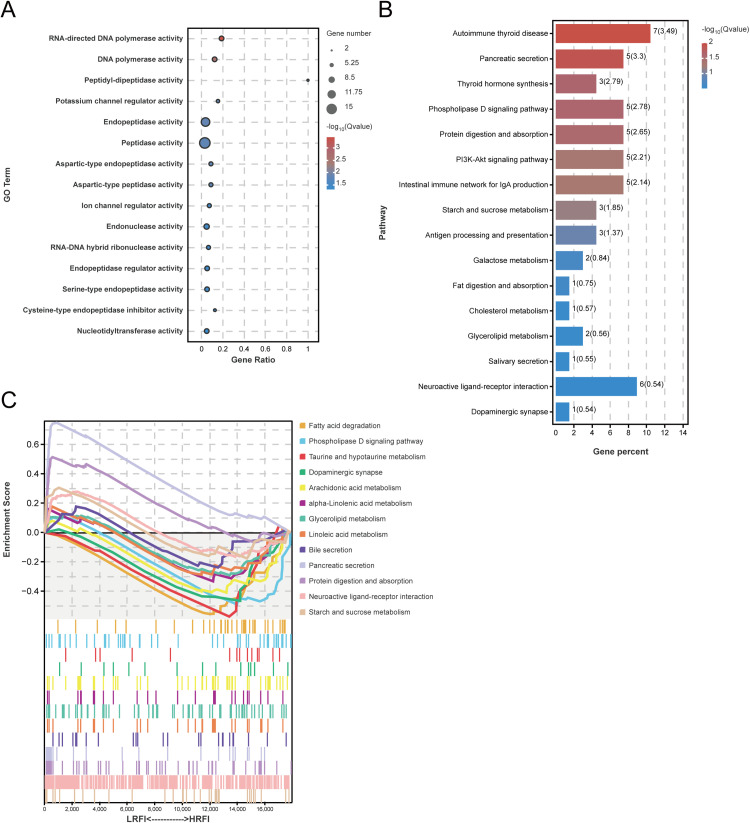
Enrichment analysis of DEGs between high and low RFI groups. A. GO enrichment analysis of DEGs. B. KEGG pathway enrichment analysis of DEGs. C. GSEA-based KEGG pathway enrichment analysis.

The 237 DEGs were significantly enriched for GO molecular functions related to proteolysis and nucleic-acid–associated catalysis, including RNA-directed DNA polymerase activity, DNA polymerase activity, peptidyl-dipeptidase activity, endopeptidase activity, aspartic-type peptidase activity, and ion channel regulator activity (Fig. 3A, Supplementary Table S6). KEGG pathway analysis highlighted processes closely tied to feed efficiency, notably digestion and absorption (protein digestion and absorption, and pancreatic secretion), lipid and carbohydrate metabolism (fat digestion and absorption, cholesterol metabolism, glycerolipid metabolism, starch and sucrose metabolism, and galactose metabolism), endocrine and membrane signaling (PI3K–Akt signaling, phospholipase D signaling, and thyroid hormone synthesis), mucosal immunity (intestinal immune network for IgA production, and antigen processing and presentation), and neural regulation (neuroactive ligand–receptor interaction, and dopaminergic synapse) (Fig. 3B, Supplementary Table S7). These enrichments converge on coordinated regulation of digestive capacity, lipid handling, and gut–brain signaling. Gene Set Enrichment Analysis (GSEA) results revealed significant enrichment of multiple KEGG pathways in the samples. The analysis further confirmed the central role of DEGs in lipid metabolism, neural regulation, and nutrient digestion and absorption. Moreover, it identified novel regulatory targets related to taurine and hypotaurine metabolism, suggesting new insights into the molecular mechanisms underlying feed efficiency (Fig. 3C).

**Fig. 4:**
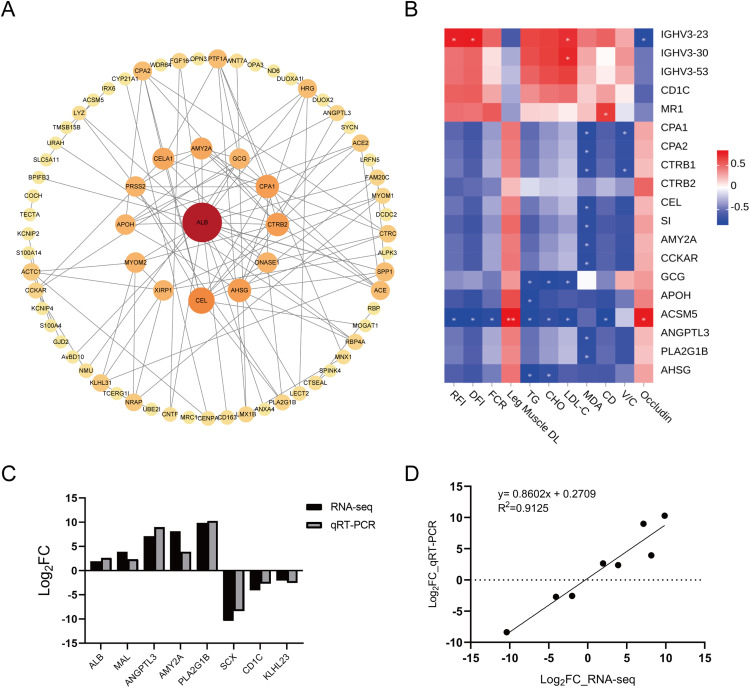
Functional validation and phenotypic correlation analysis of DEGs. A. PPI network analysis of DEGs. B. Correlation analysis between DEGs and phenotypic traits. C. Validation of randomly selected DEGs using qRT-PCR. D. Regression analysis of log₂(fold change, LRFI/HRFI) values derived from RNA-Seq and qRT-PCR data.

A PPI network constructed from DEGs identified interaction relationships among 72 genes (Fig. 4A). Using cytoHubba (MNC, MCC, Degree, and EPC ranking algorithms), eight hub genes were consistently prioritized, including *ALB, CEL, CPA1, CTRB2, AHSG, AMY2A, DNASE1* and *PRSS2*, implicating pancreatic and intestinal digestive enzymes, transport, and extracellular proteolysis as central nodes potentially mediating RFI divergence (Fig. 4A, Table 7). Collectively, the enrichment profiles and hub architecture indicate that regulation of digestion and lipid metabolism might interface with endocrine and neural pathways to support improved feed efficiency in LRFI chickens.

**Table 7:** Top 10 hub genes identified by different algorithms (MCC, MNC, Degree, and EPC).

MCC	score	MNC	score	Degree	score	EPC	score
ALB	42	ALB	11	ALB	19	ALB	32.566
CEL	30	CPA1	8	CEL	10	CTRB2	32.298
CPA1	28	CEL	8	CPA1	8	CEL	32.116
CTRB2	26	CTRB2	8	CTRB2	8	CPA1	32.033
AHSG	22	AHSG	8	AHSG	8	AMY2A	32.022
AMY2A	20	AMY2A	7	CELA1	7	CELA1	31.732
ACE	18	CELA1	6	AMY2A	7	DNASE1	31.488
DNASE1	15	DNASE1	5	DNASE1	6	AHSG	31.421
PRSS2	15	PRSS2	5	PRSS2	6	PRSS2	31.376
APOH	15	ACE	5	GCG	6	APOH	31.095

### Correlation analysis between DEGs and phenotypic traits

To further explore the biological relevance of transcriptional variation, a correlation analysis was conducted between DEGs and phenotypic traits that differed significantly between LRFI and HRFI groups. As shown in Fig. 4B, genes associated with nutrient digestion and absorption, appetite regulation, and energy metabolism exhibited negative correlations with traits such as RFI, DFI, FCR, TG, CHO, LDL-C, MDA, and CD. In contrast, genes related to inflammatory responses showed positive correlations with these phenotypic traits. These results suggest that improved feed efficiency in LRFI chickens is underpinned by transcriptional regulation promoting efficient digestion and lipid utilization, while suppressing inflammatory pathways. The integrative correlations highlight that DEGs not only reflect molecular differences between groups but also directly connect with key production traits, reinforcing their potential as biomarkers of feed efficiency.

### Serum metabolomics and differential metabolites analysis

**Fig. 5:**
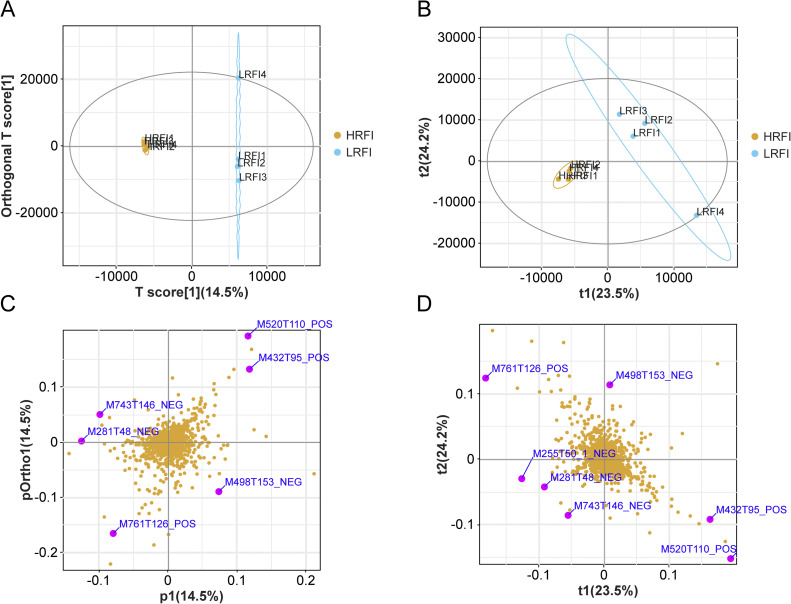
Principal component analysis of metabolites. A. Partial least squares discriminant analysis (PLS-DA) of metabolites. B. Orthogonal partial least squares discriminant analysis (OPLS-DA) of metabolites. C. PLS-DA loading plot. D. OPLS-DA loading plot.

LC–MS/MS analysis identified a total of 1,730 metabolites, including 908 in positive ion mode and 822 in negative ion mode. Multivariate statistical analyses, including PLS-DA and OPLS-DA, demonstrated clear separation between LRFI and HRFI groups with high intra-group consistency (Fig. 5A, B). The loading plots highlighted several metabolites contributing strongly to group differences, such as 2-((4R)-4-((3R,5R,9S,10S,13R,14S,17R)-3‑hydroxy-10,13-dimethylhexadecahydro-1H-cyclopentaphenanthren-17-yl)-N-methylpentanamido) ethane-1-sulfonic acid, condelphine, oleic acid, taurochenodeoxycholate, and palmitic acid (Fig. 5C, D).

**Fig. 6:**
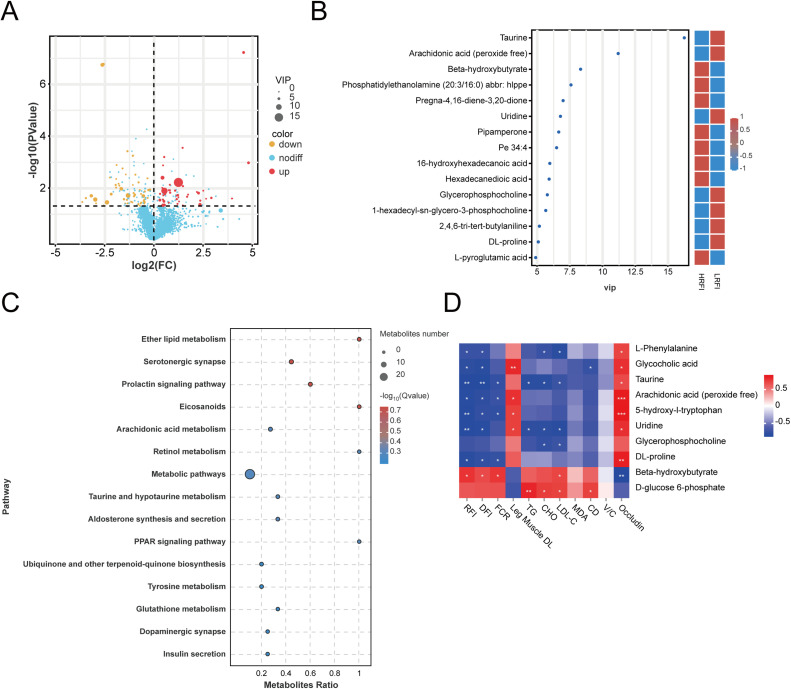
Analysis of differential metabolites between high and low RFI groups. A. Volcano plot of DEMs. B. VIP plot of the top DEMs. C. KEGG pathway enrichment analysis of DEMs. D. Correlation analysis between DEMs and phenotypic traits.

Based on VIP ≥ 1 and |log₂FC| ≥ 1, a total of 101 differentially expressed metabolites (DEMs) were identified between groups, with 54 upregulated and 47 downregulated in the LRFI group (Fig. 6A, Supplementary Table S8). The top 10 DEMs ranked by VIP values included taurine, arachidonic acid (peroxide-free), beta-hydroxybutyrate, phosphatidylethanolamine (20:3/16:0), pregna-4,16-diene-3,20‑dione, uridine, pipamperone, PE 34:4, 16-hydroxyhexadecanoic acid, and hexadecanedioic acid (Fig. 6B).

KEGG pathway enrichment analysis showed that DEMs were predominantly involved in lipid and amino acid metabolism, including ether lipid metabolism, arachidonic acid metabolism, retinol metabolism, taurine and hypotaurine metabolism, tyrosine metabolism, and glutathione metabolism. In addition, signaling pathways relevant to endocrine and neural regulation, including serotonergic synapse, prolactin signaling, dopaminergic synapse, PPAR signaling, and insulin secretion, were also significantly enriched (Fig. 6C, Supplementary Table S9). These findings highlight the central roles of lipid metabolism, amino acid turnover, and neuroendocrine regulation in feed efficiency divergence.

### Correlation analysis of DEMs with phenotypic traits

The correlation analysis showed that metabolites including L-phenylalanine, taurine, glycocholic acid, arachidonic acid (peroxide-free), uridine, glycerophosphocholine, and DL-proline were negatively associated with RFI, DFI, FCR, serum lipid indices (TG, CHO, and LDL-C), oxidative stress marker MDA and CD. Conversely, these metabolites were positively correlated with leg muscle drip loss and duodenal *Occludin* expression (Fig. 6D). By contrast, beta-hydroxybutyrate and D-glucose 6-phosphate exhibited opposite associations. Collectively, these results suggest that specific metabolites, particularly taurine, L-phenylalanine, and uridine, may serve as biomarkers of enhanced feed efficiency, linking reduced feed intake to improved lipid metabolism, lower oxidative stress, and better intestinal integrity.

### Integrated analysis of transcriptomic and metabolomic data

**Fig. 7:**
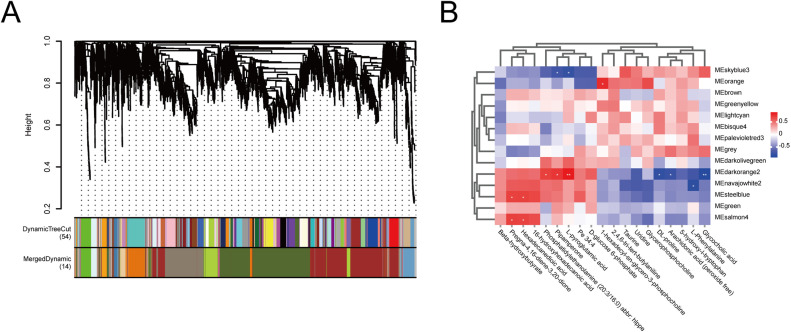
Integrated analysis of differentially expressed genes and metabolites. A. WGCNA identifying gene modules associated with RFI. B. Heatmap showing correlations between gene co-expression modules and differential metabolites.

To further elucidate the gene regulatory mechanisms underlying RFI, a Weighted Gene Co-expression Network Analysis (WGCNA) was performed, identifying 14 distinct gene co-expression modules (Fig. 7A). Among them, the skyblue3 module showed a significant positive correlation with the LRFI group. According to the module–metabolite correlation heatmap, transcript accumulation in the skyblue3 module was positively correlated with several metabolites, including taurine, uridine, glycerophosphocholine, DL-proline, arachidonic acid (peroxide free), 5‑hydroxy-L-tryptophan, L-phenylalanine, and glycocholic acid (Fig. 7B). These metabolites are mainly associated with appetite regulation and energy metabolism. Key DEGs within the skyblue3 module included *CPA2, CPA1, SI, CCKAR, AMY2A, AHSG, CTRC, CTRB1, CELA1, ANGPTL3, PLA2G1B, CEL*, and *APOH*, suggesting that this module may play a critical role in modulating RFI through pathways related to digestion, nutrient absorption, and metabolic regulation.

**Fig. 8:**
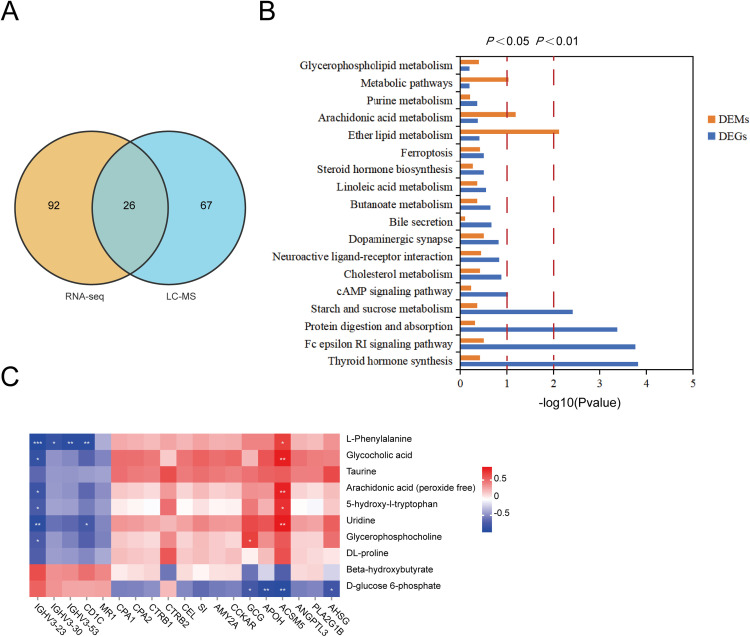
Identification of key metabolic pathways affecting RFI. A. Venn diagram of enriched KEGG pathways shared between DEGs and DEMs. B. Commonly enriched pathways of DEGs and DEMs. C. Correlation analysis between DEGs and DEMs.

To determine the correlation between transcriptomic and metabolomic data, 26 shared KEGG pathways were identified between the two datasets (Fig. 8A). These pathways primarily involved protein and lipid metabolism as well as central nervous system-related functions (Fig. 8B). Notably, DEGs and DEMs within these pathways exhibited significant correlations (Fig. 8C). In summary, the integrated omics analysis uncovered a regulatory network involving digestive enzymes, lipid transport, amino acid metabolism, and gut–brain signaling, providing potential biomarkers for improving feed efficiency in local chicken breeds.

## Discussion

Feed efficiency plays a critical role in poultry production, directly influencing farming profitability and environmental sustainability. Compared to commercial breeds, indigenous chicken breeds generally exhibit lower feed efficiency. Therefore, elucidating the genetic mechanisms underlying RFI in local breeds and improving their feed efficiency has become a key breeding objective.

Studies have shown that the core principle of improving feed efficiency through selection for RFI lies in the animal’s ability to reduce feed intake while maintaining production performance (Aggrey and Rekaya, 2013). In this study, we analyzed the correlation between RFI and various production traits in Tianchang Sanhuang chickens. The results revealed a highly significant positive correlation between RFI and feed intake, whereas no significant associations were found between RFI and average daily gain, body weight, or egg production. The further comparison analysis demonstrated that the feed intake of the LRFI group was approximately 68% of that in the HRFI group. These findings suggest that selection based on RFI can effectively reduce feed consumption without adversely affecting production traits, which is consistent with previous studies in commercial laying hens, commercial broilers, and native chicken breeds (Dadfar et al., 2023; Zhao et al., 2022; Zou et al., 2023). We also evaluated meat quality traits in both groups, and the results showed that the redness (a*) value of the breast muscle and the drip loss of leg muscle were significantly negatively correlated with RFI. This may be related to higher physical activity in the HRFI group (Lefaucheur et al., 2011), suggesting that selection based on RFI may have some impact on meat quality. Additionally, serum biochemical and antioxidant analyses revealed that TG, CHO, LDL-C, and MDA were significantly positively correlated with RFI. These results align with findings reported by Bai et al. (2022) in small-sized meat ducks and by Metzler-Zebeli et al. (2019) in broilers. The elevated triglyceride levels indicate greater fat accumulation, potentially resulting from an imbalance between energy intake and expenditure in HRFI chickens. Cholesterol levels reflect lipid metabolism and dietary fat intake in poultry (Shafiq et al., 2022). LDL-C is primarily responsible for transporting cholesterol from the liver to peripheral tissues, maintaining membrane integrity and supporting cellular signaling and transport functions (Yeang et al., 2022); thus, LDL-C indirectly reflects how cholesterol metabolism influences feed conversion efficiency. MDA, a byproduct of lipid peroxidation, is commonly used as a biomarker for oxidative stress and cellular damage. Excessive MDA concentrations may indicate an overaccumulation of body fat or impaired lipid metabolism, both of which could contribute to reduced feed efficiency. These results suggest that differences in RFI may be associated with increased fat storage, imbalanced lipid metabolism, and elevated oxidative stress—all of which collectively impact feed efficiency.

The duodenum plays a crucial role in the digestive system of chickens, serving not only as a primary site for food digestion but also as a key region for nutrient absorption. Its functional efficiency is closely associated with intestinal morphology, particularly VH, CD, and V/C (Xie et al., 2020). In the present study, we found that the CD of the duodenum in the LRFI group was significantly shallower than that of the HRFI group, suggesting an enhanced ability to digest and absorb nutrients through improved intestinal morphology. This observation is consistent with findings by Metzler et al. (2017) in pigs, where lower RFI pigs exhibited reduced duodenal crypt depth, potentially reflecting a slower epithelial cell proliferation rate. Given that intestinal epithelial cell turnover is energy-intensive, the shallower crypts in low-RFI animals may indicate reduced energy expenditure for gut maintenance, thereby improving overall feed efficiency. Moreover, the expression level of the tight junction protein *Occludin* was significantly higher in the LRFI group. *Occludin* is a key component of intestinal tight junctions and plays a critical role in regulating paracellular permeability (Kuo et al., 2022). This finding suggests that the intestinal barrier function in the LRFI group is likely more intact, which may help prevent harmful substances from translocating across the intestinal epithelium into systemic circulation, thereby reducing the risk of inflammation and immune activation (Park et al., 2024). Notably, the integrity of the intestinal barrier is not only vital for immune regulation but may also indirectly improve nutrient absorption by maintaining a favorable gut microenvironment. A healthy intestinal mucosa typically exhibits a higher V/C ratio, which directly correlates with nutrient absorption surface area and epithelial renewal efficiency (La et al., 2023) Animals in the low-RFI group may benefit from better gut barrier function, which reduces energy expenditure on immune responses and allows a greater portion of energy to be allocated to growth and metabolism potentially explaining their superior feed efficiency.

Transcriptomic analysis of the duodenum from chickens with divergent RFI values revealed that DEGs were primarily enriched in pathways related to immune responses, digestive metabolism, and the nervous system. It is widely accepted that during immune activation, the body’s energy requirements for maintenance increase, and nutrient allocation shifts from productive processes toward immune-related functions. This redistribution of nutrients can reduce feed efficiency in animals (Wang et al., 2025). Similarly, Yang et al. (2020a) reported that in Chinese indigenous chicken breeds, immune-related DEGs and pathways were downregulated in the LRFI group. Studies in beef cattle have also found that animals with higher feed efficiency exhibit weaker inflammatory responses (Keogh et al., 2024).

In the present study, several immune-related DEGs immunoglobulin heavy-chain variable regions 3-23 (*IGHV3-23*), *IGHV3-30, IGHV3-53, CD1C*, and *MR1* were enriched in pathways such as autoimmune thyroid disease, primary immunodeficiency, and antigen processing and presentation. All of these genes showed a consistent downregulation trend in the LRFI group. Immunoglobulins are critical components of the immune system, involved in both innate and adaptive immunity. The *IGHV3-23, IGHV3-30*, and *IGHV3-53* genes encode *IGHV*, which are major contributors to antibody diversity. These *IGHV3* genes play pivotal roles in the diversity and specificity of B cell receptors (BCRs), essential for recognizing a wide array of antigens. Notably, all three *IGHV* genes were significantly correlated with the metabolite arachidonic acid (peroxide free) in this study. Arachidonic acid (peroxide free) is a polyunsaturated fatty acid that serves as a precursor for various bioactive lipid mediators, such as prostaglandins, leukotrienes, and thromboxanes. These mediators are essential in regulating inflammation, immune responses, and vascular function (Astudillo et al., 2009). *CD1C*, which encodes a non-polymorphic *MHC* class I-like molecule, differs from classical *MHC* I proteins by presenting lipid and glycolipid antigens to T cells—particularly natural killer T (NKT) cells and subsets of helper T cells (Th1 and Th17). *CD1C* plays a crucial role in initiating and modulating immune responses, especially in defense against pathogens like bacteria, viruses, and parasites (Bao et al., 2024). The downregulation of these immune-related genes in the LRFI group suggests that reduced immune activation may help conserve energy, allowing more nutrients to be allocated toward growth and production rather than immune defense. This energy-saving mechanism could be a contributing factor to the improved feed efficiency observed in low-RFI chickens.

In the gastrointestinal tract, the pancreas and duodenum play a vital role in nutrient absorption through the secretion of digestive enzymes and hormones. In this study, several genes associated with pancreatic enzyme synthesis were significantly upregulated in the duodenum of LRFI chickens. They included genes encoding lipases (*PLA2G1B*), amylases (*AMY2A* and *SI*), and proteases (*CPA1, CPA2, CEL, CTRB1*, and *CTRB2*). *PLA2G1B* belongs to the secretory phospholipase A2 (sPLA2) family and has been reported to act as a regulator of lipoprotein particle size, notably influencing the expression of apolipoprotein *APOB* (Fu et al., 2020). In humans, genetic polymorphisms in *PLA2G1B* have been significantly associated with obesity. The gene encodes phospholipase A2 group 1B, a key lipase involved in lipid hydrolysis, catalyzing the conversion of triglycerides into monoglycerides and free fatty acids (Cash et al., 2011). The elevated mRNA expression of *PLA2G1B* in LRFI chickens suggests that these birds may enhance lipid hydrolysis and fatty acid release, thereby improving their ability to utilize dietary lipids more efficiently. This increased digestive efficiency could be one of the mechanisms contributing to the superior feed efficiency observed in LRFI chickens.

*AMY2A* and SI were significantly enriched in the starch and sucrose metabolism pathway. The *AMY2A* gene encodes α-amylase, which plays a crucial role in carbohydrate and glycogen metabolism (Hariharan et al., 2021). This gene has been shown to influence chicken growth, carcass traits, and feed efficiency by promoting dietary starch digestion and carbohydrate metabolism (Zhang et al., 2021). *AMY2A* indirectly affects glucose metabolism by breaking down starch into glucose, which is subsequently phosphorylated to form D-glucose 6-phosphate. This metabolite serves as a central intermediate in glycolysis, gluconeogenesis, and the pentose phosphate pathway, making it critical for energy metabolism in the body (Zhang et al., 2025). Consistent with this, metabolomic studies of milk from ewes with different RFI levels have also shown that differential metabolites are significantly enriched in glycolysis, gluconeogenesis, and fructose metabolism pathways (Marina et al., 2024). In the present study, D-glucose 6-phosphate was upregulated in the HRFI group, indicating an enhanced energy metabolic state, which may be associated with the higher metabolic rate observed in HRFI chickens. Meanwhile, *SI* (sucrase-isomaltase) is the most important disaccharidase in the small intestine and responsible for hydrolyzing sucrose and starch-derived carbohydrates into monosaccharides for absorption (Gericke et al., 2016). In cases of carbohydrate malabsorption, SI deficiency can lead to reduced body weight due to inefficient energy utilization (Andersen et al., 2022). By regulating glucose absorption, SI may also indirectly influence the levels of D-glucose 6-phosphate, linking its expression to broader aspects of energy metabolism and feed efficiency.

Genes significantly enriched in the protein digestion and absorption pathway include *CPA1, CPA2, CTRB1*, and *CTRB2*. Carboxypeptidase A (CPA) is synthesized and secreted in the form of a zymogen and becomes activated upon cleavage by trypsin. It functions through its zinc carboxypeptidase domain to degrade amino acid residues at the carboxy-terminal end of proteins (Vendrell et al., 1990). Previous studies have shown that *CPA1* mRNA expression is significantly elevated in broilers with improved intestinal digestive capacity after supplementation with moringa oleifera leaf meal (Khalid et al., 2022). Furthermore, *CPA1* and *CPA2* have been identified as biomarkers for pancreatic injury, with their expression markedly reduced in mice experiencing pancreatic damage (Vlasakova et al., 2023). Both *CPA1* and *CPA2* facilitate protein digestion, and were found to be significantly positively correlated with the metabolite L-Phenylalanine in the present study. L-Phenylalanine is involved in protein synthesis and serves as a precursor for intestinal hormones that induce satiety, which can suppress appetite. Feng et al. (2019) reported that L-Phenylalanine stimulates the release of cholecystokinin (CCK) and glucose-dependent insulinotropic polypeptide (GIP) in the duodenum, thereby regulating feed intake and energy metabolism. Given its dual role in appetite and metabolism regulation, L-Phenylalanine has the potential to serve as a key biomarker for identifying RFI. *CTRB1* and *CTRB2* encode chymotrypsinogen B1 and B2, precursors of digestive enzymes secreted by the pancreas. They are activated in the small intestine and participate in the hydrolysis of dietary proteins and peptides. In addition to aiding protein digestion, they may protect animals from pancreatitis by regulating the degradation of trypsinogen (Mosztbacher et al., 2020). Similar to CPA enzymes, *CTRB1* and *CTRB2* contribute to the release of amino acids from dietary proteins, potentially influencing the availability of L-Phenylalanine for metabolic processes. Overall, these findings suggest that low RFI chickens may improve feed efficiency by upregulating mRNA expression of pancreatic genes involved in digestive enzyme production, thereby enhancing nutrient absorption and utilization in the intestine.

The gut-brain axis is a complex bidirectional communication network involving interactions between the brain and the gastrointestinal tract, with nervous, endocrine, and immune systems all playing important roles. Among these, the neuroactive ligand-receptor interaction pathway is particularly critical in transmitting signals within this axis (Neff et al., 2021). In this study, cholecystokinin A receptor (*CCKAR*) and *GCG* were identified as significantly enriched differentially expressed genes within this pathway. *CCKAR*, a member of the cholecystokinin receptor family, has been reported to be closely associated with appetite regulation (Dunn et al., 2013). *CCK* is a gastrointestinal hormone that induces gallbladder contraction and promotes the secretion of pancreatic enzymes. The combined action of endogenous *CCK* and its receptor *CCKAR* can activate satiety signals in the central nervous system, thereby regulating food intake and energy metabolism (Jiao et al., 2022). Our results showed that *CCKAR* was significantly upregulated in the LRFI group of Tianchang Sanhuang chickens, consistent with previous findings in native breeds (Yi et al., 2018), suggesting a key role for *CCKAR* in regulating feeding behavior and energy balance. The upregulation of *CCKAR* may help enhance satiety signaling and improve feed efficiency. At the metabolite level, 5‑hydroxy-L-tryptophan (5-HTP), a precursor of the neurotransmitter serotonin (5-HT), was found to align with *CCKAR* expression patterns in this study. 5-HT is widely present in the central nervous system and gastrointestinal tract, where it regulates various physiological functions such as mood, sleep, and appetite, and enhances the sensation of satiety by acting on multiple brain regions. Studies have shown that supplementation with 5-HTP significantly increases melatonin levels by enhancing pineal 5-HT and its metabolite N-acetylserotonin (Sugden et al., 1985). Melatonin plays an important role in reproductive regulation, promoting follicle development, increasing egg production, and improving egg weight (Jia et al., 2016; Sundaresan et al., 2009). These findings suggest that LRFI chickens may regulate satiety and reproductive performance through the gut-brain axis via the 5-HT-melatonin pathway, thereby improving feed conversion efficiency. Moreover, the metabolite taurine, which was also enriched in this pathway, showed a significant negative correlation with RFI, DFI, and FCR in this study, indicating its potential role in regulating feed efficiency. Taurine is involved in bile acid conjugation, neural signaling, membrane stabilization, and cholesterol metabolism (Prakashan et al., 2024). Dietary taurine supplementation in broilers can enhance antioxidant capacity and regulate lipid metabolism, thereby reducing oxidative stress and lipid deposition and ultimately improving feed efficiency (Han et al., 2020). Metzler-Zebeli et al. (2019) reported that in a serum metabolomics analysis of broilers taurine reduces RFI by influencing feed intake. Finally, the *GCG* gene encodes proglucagon, which is enzymatically cleaved to produce glucagon. This hormone promotes glycogen breakdown in the liver, inhibits glycogen synthesis, enhances gluconeogenesis and glycolysis, and indirectly facilitates lipid mobilization, while also playing a key role in amino acid metabolism (Sandoval and D'Alessio, 2015). In summary, LRFI chickens may achieve higher feed efficiency by modulating central satiety, energy metabolism, and reproductive function through coordinated regulation of neurotransmitters, hormone receptors, and key metabolites within the gut-brain axis.

Among the mRNAs related to lipid metabolism, several genes showed significant differences in expression levels, including *ANGPTL3, APOH, ACSM5*, and *AHSG*, which were primarily enriched in biological pathways such as cholesterol metabolism, bile secretion, butanoate metabolism, and linoleic acid metabolism. Angiopoietin-like protein 3 (*ANGPTL3*) is a secretory protein that regulates lipid, glucose, and energy metabolism. Shimamura et al. (2003) found that liver-specific *ANGPTL3* can act directly on adipocytes, stimulating lipolysis and the release of free fatty acids and glycerol. *ANGPTL3* modulates fat metabolism by inhibiting lipases and promoting lipid breakdown. Studies on different adipose tissues in chicks have identified *ANGPTL3* as a gene associated with adipose tissue deposition (Wei et al., 2024). Our study found that *ANGPTL3* expression was significantly upregulated in the LRFI group, and its expression level was negatively correlated with TG and LDL-C, indicating enhanced lipid utilization. The *APOH* gene encodes apolipoprotein H, which is involved in lipid metabolism, coagulation, and inflammation. It has been shown to be associated with triglyceride metabolism, and variations in *APOH* can influence TG levels (Mather et al., 2016). In this study, *APOH* showed a significant negative correlation with TG, suggesting that *APOH* may reduce fat deposition in LRFI chickens by promoting TG metabolism. Glycocholic acid, which is involved in the cholesterol metabolism pathway, was positively correlated with *APOH*, indicating that *APOH* may indirectly influence bile acid synthesis by regulating cholesterol and triglyceride metabolism. As a type of bile acid, glycocholic acid aids in the digestion and absorption of fats and fat-soluble substances. Studies have shown that dietary supplementation with glycocholic acid in feed improves growth performance and mitigates liver and intestinal damage caused by high pectin diets (Yao et al., 2024). *ACSM5*, a member of the medium-chain acyl-CoA synthetase family, participates in acyl-CoA metabolism and fatty acid biosynthesis. Its differential expression in liver and adipose tissues suggests a potential role in fatty acid metabolism and energy storage. In this study, *ACSM5* was significantly negatively correlated with TG, CHO, and LDL-C. Other research has shown that *ACSM5* expression is associated with fatty acid composition in adipose tissue, indicating its involvement in regulating fat metabolism and fatty acid profiles, thereby potentially affecting livestock growth efficiency (Revilla et al., 2018). Alpha-2-HS-glycoprotein (*AHSG*) is a plasma glycoprotein synthesized and secreted by hepatocytes, involved in bone metabolism, inflammatory processes, and insulin resistance (Guarneri et al., 2013). *AHSG* is considered a candidate gene associated with fat deposition and metabolic regulation (Lavebratt, 2005). In this study, *AHSG* expression in the duodenum was significantly higher in LRFI Tianchang Sanhuang chickens compared to the HRFI group. In studies on abdominal fat of broilers with different feed efficiencies, *AHSG* has been found to affect fat deposition by regulating adiponectin, thereby influencing glucose uptake and lipid oxidation in adipocytes (Zhuo et al., 2015). Similarly, Zhu et al. (2023) found a significant negative correlation between *AHSG* protein levels and RFI in Yorkshire pigs, further supporting *AHSG* as a candidate gene associated with high feed efficiency. Albumin (*ALB*), identified as a core gene in this study due to its high correlation with other DEGs, is involved in the thyroid hormone synthesis pathway. The upregulation of *ALB* may enhance the transport efficiency of nutrients, improve their tissue utilization, and reduce metabolic waste, thereby lowering the energy requirement for maintenance.

## Conclusions

This study demonstrates that selection for low RFI in Tianchang Sanhuang chickens improves feed efficiency without compromising growth or production traits. By integrating transcriptomic and metabolomic analyses, we identified novel candidate genes (*ACSM5, AHSG, CTRB1, PLA2G1B, AMY2A, CPA1,* and *CCKAR*) and pathways related to digestive enzyme activity, lipid metabolism, and gut–brain signaling. Importantly, gene–metabolite correlations revealed strong associations between transcriptional regulation and key metabolites such as taurine, uridine, L-phenylalanine, D-glucose 6-phosphate and 5‑hydroxy-L-tryptophan, which are linked to appetite regulation, energy metabolism, and oxidative stress resistance. These findings implicate that RFI divergence is mediated by coordinated gene–metabolite networks rather than isolated traits. Collectively, this study advances the understanding of feed efficiency in local chicken breeds and proposes potential molecular biomarkers for precision breeding strategies.

## Data availability

The transcriptome sequencing data were deposited in the Sequence Read Archive (SRA) database (https://www.ncbi.nlm.nih.gov/sra) of NCBI under the BioProject accession numbers PRJNA1266125 and SAMN 48665732 to 48665739, and the SRA project accession numbers SRR 33659432 to 33659439.

## Disclosures

The authors declare no conflicts of interest.

## Declaration of generative AI and AI-assisted technologies in the writing process

During the preparation of this work, the authors only used these technologies to improve readability and language.

## CRediT authorship contribution statement

**Jiale Li:** Writing – original draft, Software, Formal analysis, Data curation, Conceptualization. **Shenghe Li:** Writing – original draft, Visualization, Resources, Project administration, Methodology, Conceptualization. **Mengmeng Zhuansun:** Writing – original draft, Supervision, Investigation, Data curation. **Xinyu Liu:** Validation, Methodology, Investigation, Data curation. **Tao Jin:** Visualization, Validation. **Kefeng Yang:** Investigation, Data curation. **Man Ren:** Supervision, Resources, Project administration. **Erhui Jin:** Software, Funding acquisition, Conceptualization. **Xiaojin Li:** Validation, Resources, Project administration. **Mengmeng Jin:** Writing – review & editing, Project administration. **Chunfang Zhao:** Writing – review & editing, Project administration, Funding acquisition, Conceptualization.

## Disclosures

The authors declare that they have no known competing financial interests or personal relationships that could have appeared to influence the work reported in this paper.

## Appendix Supplementary materials

Image, application 1

## Footnotes

Supplementary material associated with this article can be found, in the online version, at doi:10.1016/j.psj.2025.105912.
