# Supplementary material for: Integrated transcriptomic and metabolomic analysis reveals key regulatory genes and pathways associated with feed conversion efficiency in Tianchang Sanhuang chicken
Source: Poult Sci. 2025 Sep 27;104(12):105912. doi: 10.1016/j.psj.2025.105912 (PMC12523079; doi:10.1016/j.psj.2025.105912)
Supplement: Supplementary file 1 [file mmc1.zip › Supplementary Table S1.docx]

**Table S1 Feed Composition and Nutritional Value**

| Ingredient | Content (%) | Nutrient Level | Content (%) |
| --- | --- | --- | --- |
| Corn | 56.00 | Metabolizable Energy (MJ/kg) | 10.60 |
| Soybean Meal | 19.40 | Crude Protein | 15.50 |
| Wheat Bran | 15.20 | Crude Fiber | 3.50 |
| Fish Meal | 3.00 | Lysine | 0.90 |
| Calcium Bicarbonate | 0.30 | Methionine | 0.30 |
| Limestone | 1.10 | Threonine | 0.67 |
| Premix | 5.00 | Calcium | 0.95 |
| Total | 100.00 | Total Phosphorus | 0.45 |

Note:

1.The premix provided the following per kilogram of diet: VA 10,000 IU, VD_3_ 3,000 IU, VE 20 IU, VK 3 IU, VB_12_ 0.025 mg, VB_1_ 1.8 mg, VB_2_ 5.0 mg, biotin 0.04 mg, folic acid 0.6 mg, VB_5_ 12 mg, VB_3_ 12 mg, VB_6_ 3.75 mg, Mn 100 mg, Zn 75 mg, Fe 80 mg, Cu 8 mg, I 0.15 mg, and Se 0.10 mg.

2.Metabolizable energy is a calculated value; all other values are based on actual measurements.
