# Supplementary material for: Integrated transcriptomic and metabolomic analysis reveals key regulatory genes and pathways associated with feed conversion efficiency in Tianchang Sanhuang chicken
Source: Poult Sci. 2025 Sep 27;104(12):105912. doi: 10.1016/j.psj.2025.105912 (PMC12523079; doi:10.1016/j.psj.2025.105912)

**Figure S1 Histomorphological analysis of the duodenum in Tianchang Sanhuang chickens with high residual feed intake**


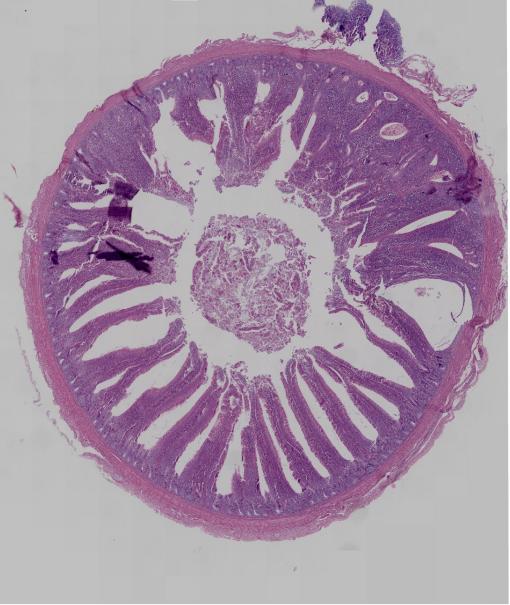


**Figure S2 Histomorphological analysis of the duodenum in Tianchang Sanhuang chickens with low residual feed intake**


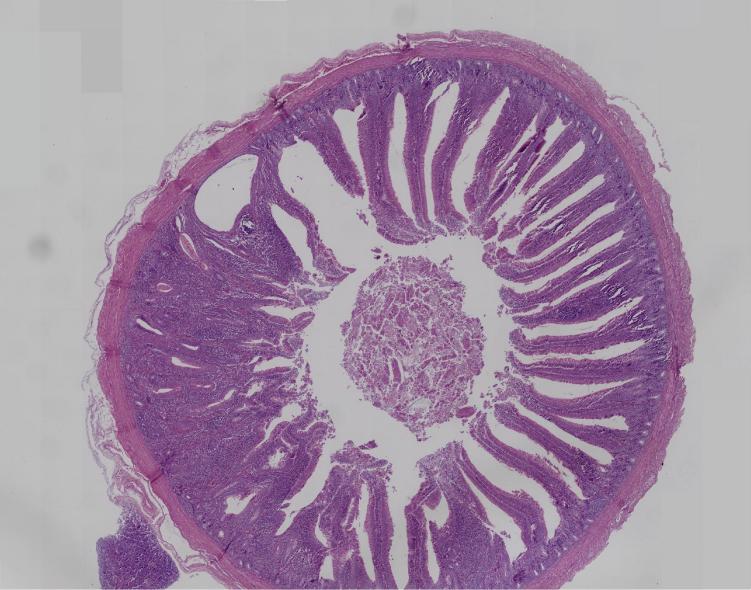

Supplement: Supplementary file 1 [file mmc1.zip › Supplementary.docx]
